# Genetic Polymorphisms of CCL22 and CCR4 in Patients with Lung Cancer

**Published:** 2014-07

**Authors:** Nasrollah Erfani, Ahmadi-Sina Nedaei Ahmadi, Mohammad Ali Ghayumi, Zahra Mojtahedi

**Affiliations:** 1 Shiraz Institute for Cancer Research, School of Medicine, Shiraz University of Medical Sciences, Shiraz, Iran;; 2 Student Research Committee, School of Medicine, Shiraz University of Medical Sciences, Shiraz, Iran;; 3 Department of Internal Medicine, Faghihi Hospital, Shiraz University of Medical Sciences, Shiraz, Iran

**Keywords:** Polymorphism, Genetic, Lung Neoplasms, Chemokine CCL22, CCR4

## Abstract

**Background:** An association between lung cancer and chemokines has been advocated in the recent years. This study aims at investigating the association between lung cancer and 16C/A single nucleotide polymorphism (SNP) (rs. 4359426) in C-C motif chemokine 22 (CCL22) as well as C1014T SNP (rs. 2228428) in C-C chemokine receptor type 4 (CCR4), which serves as the receptor for CCL22.

**Methods: **Genotyping was performed in 148 lung cancer patients and 148 normal controls using Polymerase Chain Reaction-Restriction-Fragment Length Polymorphism (PCR-RFLP). The data were verified by direct automated sequencing.

**Results: **Frequencies of CC, CA and AA genotypes of 16C/A SNP in CCL22 gene were 112 (75.7%), 33 (22.3%) and 3 (2.0%) in patients, and 119 (80.4%), 24 (16.2%) and 5 (3.4%) in controls respectively. No significant differences were observed in genotype frequencies at this position between cases and controls (P=0.34). Moreover, there was no significant association between CCL22 polymorphism and types of lung cancer in patients. The distribution of CC, CT and TT genotypes of C1014T SNP in CCR4 gene, was 76 (51.4%), 60 (40.5%) and 12 (8.1%) in patients, and 80 (54.1%), 49 (33.1%) and 19 (12.8%) in controls respectively. No statistically significant differences were observed in genotypes frequencies of CCR4 gene between patients and controls (P=0.24). The genotype inherited by patients observed not to be associated with the type of lung cancer (P>0.05).

**Conclusion: **Results reveal that CCL22 gene polymorphism at position 16C/A and CCR4 gene polymorphism at position C1014T, appear not to be associated with susceptibility to lung cancer.

## Introduction

Lung cancer is one of the most life-threatening types of cancer in both genders.^1^ With industrial development and increased tobacco smoking in Iran, the rate of this cancer has been increased in recent decade. Lung cancer has become the third most frequent cancer in both genders accounting for 4% of the country’s health costs.^2^^,^^3^ Complex interactions between genetic, hormonal, behavioral and environmental factors have been implicated in the development of lung cancer.^1^^,^^4^ Lung cancer is histologically classified into small cell lung cancer (SCLC, accounting for 15-25% of all lung cancers) and non-small cell lung cancer (NSCLC, 75-85%). NSCLC is the main type of lung cancer and is then sub-divided into squamous cell carcinoma, adenocarcinoma and large cell carcinoma.

Chemokines are chemotactic cytokines that mediate cellular trafficking, leukocyte maturation, homing of lymphocytes and the development of lymphoid tissues. Up to now more than 50 different chemokines and 20 chemokine receptors have been introduced in humans.^5^ The gene for C-C motif chemokine 22 (CCL22) is located on the short arm of chromosome 16(q16) and encodes for a class of cys-cys (CC) chemokines. The common characteristic of CC chemokines is that they have two consecutive cysteines in their protein structure.^6^ The cytokine that is produced by this gene is chemo-attractant for monocytes, dendritic cells, natural killer cells and T lymphocytes. C-C chemokine receptor type 4 (CCR4) protein is a G-protein coupled receptor that acts as the receptor for CCL22.^6^ CCL22 is mainly produced by the immune cells.^6^^,^^7^ Expression of this chemokine and its receptor, however, was reported in malignancies.^8^^-^^10^ CCR4 has been reported to be preferably expressed on the surface of T helper type 2 (Th2) and Regulatory T (Treg) lymphocytes, and recruits these cells to the site of tumor.^11^^-^^13^ Several polymorphisms have been identified in CCL22 and CCR4 genes, some of which with the functional effects on the susceptibility to immunological diseases and malignancies.^14^^-^^16^

The aim of the present study was to investigate the association of 16C/A SNP (reference SNP no. 4359426) in CCL22 gene (i.e. causes a 2-aspartate to 2-alanine substitution in the CCL22 protein), as well as C1014T SNP (reference SNP no. 2228428) in CCR4 (i.e. causes a silent mutation at position 338 in CCR4 protein [Tyrosine residue]) with an increased risk of developing lung cancer. The association of the genotypes with the subtypes of lung cancer (Small cell lung cancer and Non-small cell lung cancer) was also investigated. 

## Patients and Methods

In a case-control study, 148 patients with lung cancer and 148 healthy individuals were recruited. The inclusion criteria for the patients were the primary diagnosis of lung cancer based on clinical work-up and definitive pathological diagnosis. The control group consisted of normal healthy individuals with no history of malignancies or autoimmune diseases in their immediate relatives. They were matched with the case group according to age and gender. 

This study was approved by the Medical Ethics Committee of Shiraz University of Medical Sciences. Participants were informed that blood samples would be used for a research project and informed consent was obtained. 5mL venous blood sample (with EDTA 10%) was obtained from each subject followed by DNA extraction of each sample by salting-out method. Genotyping was performed using Polymerase Chain Reaction-Restriction Fragment Length Polymorphism (PCR-RFLP) method. A 215 bp band around CCL22 16C/A locus was amplified using primer pairs 5’-TGGGAGGTAGTTCTTCTTTTGA-3’ (forward) and 5’-CCACAGCAAGGAGGACGA-3’ (reverse).^16^ PCR was performed with a start denaturation of 94°C for 5 minutes followed by 35cycles of denaturation (94°C, 30 seconds), annealing (64°C, 30 seconds) and extension (72°C, 30 seconds). The reaction was terminated with a final extension at 72°C for 5 minutes. The PCR products were treated with restriction endonuclease *Mbo*I overnight (CinnaGen, Iran). Three different genotypes were identified after agarose gel electrophoresis and gel staining with GelRed (Biotium, USA) as published previously.^17^ The genotyping data was verified by automated DNA sequencing using BigDey terminator chemistry kit (ABI, USA) and 310 genetic analyzer (ABI, USA) as previously published.^17^

**Figure 1:**
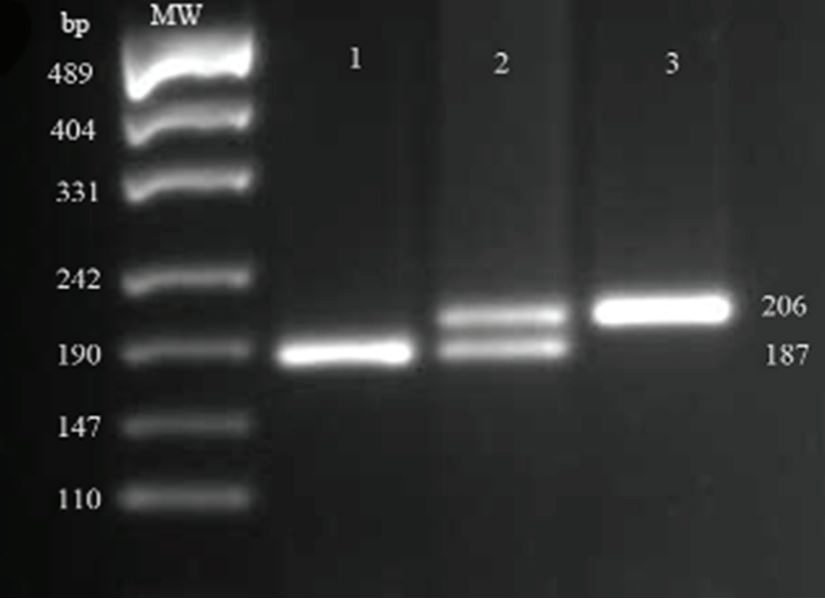
Results of Restriction Fragment Length Polymorphism (RFLP) using *RsaI *for detection of 1014C/T genetic variation in CCR4 gene on agarose gel 2.5%. TT homozygote, CT heterozygote, and CC homozygote samples have been run in lane 1, 2, and 3, respectively. MW: molecular size marker.

**Figure 2:**
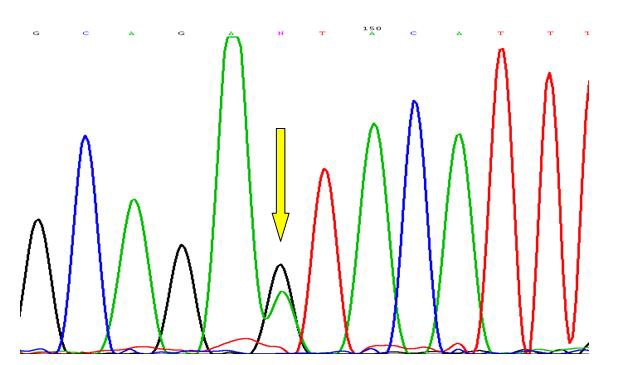
Verifying the heterozygote genotype at position 1014C/T in CCR4 gene (rs2228428) by direct automated sequencing. The heterozygote peak is pointed by arrow.

Primer pairs 5’-TGTGGGCTCCTCCAAATGTA-3’ (forward: 1011TwG, mismatch primer) and 5’-TGTAAGCCTTCCTCCTGACA-3’ (reverse)^18^ were used to amplify a 206 bp band around CCR4 C1014T SNP. PCR protocol was the same as CCL22 16C/A position except for the annealing temperature which was performed at 48°C for 30 seconds. The amplified products were digested overnight with *Rsa*I restriction enzyme (Fermentas, Lithuania). Different genotypes were then identified after gel electrophoresis and staining as illustrated in figure 1. The data of CCR4 genotyping was verified by automated DNA sequencing as mentioned above (figure 2).

Statistical analysis was performed using SPSS software version 11.5 (SPSS Inc, Chicago, IL, USA) and P value for statistical significance was defined as P<0.05. The test for Hardy-Weinberg Equilibration was performed using Arlequin software package, version 3.1.^19^

## Results

Data from 148 patients clinically and pathologically confirmed to have lung cancer were analyzed, including 116 (out of 145, 80%) non-small cell lung cancer individuals and 29 (out of 145, 20%) small cell lung cancer patients. Three patients were known cases of lung cancer but their tumor types were not available in their medical files. 83% of cases, as well as controls, were male and 17% were female. The age of the patients ranged from 30 to 85 years with a mean of 64.4 years. The most crowded age group was those over 70s with 32.4% of all cases. Table 1 illustrates the characteristics of the lung cancer patients and healthy controls.

**Table 1:** Characteristics of the lung cancer patients and healthy controls

**Group**	**Age (mean±SD)** **n=148**	**Sex**	**Tumor type**
Controls	63.5 y±10.6 Min: 30, Max: 85	M: 123 (83.1 %) F: 25 (16.9 %)	NA
Patients	64.4 y±11.9 Min: 30, Max: 85	M: 123 (83.1 %) F: 25 (16.9 %)	SCLC: 29 (20%) NSCLC: 116 (80%) Unknown: 3٭

*Three patients were known lung cancer patients with the tumor type not available in their medical files. NA: Not applicable; Max: Maximum; Min: Minimum; SCLC: Small cell lung cancers; NSCLC: Non-small cell lung cancers; M: Male; F: female; SD: Standard deviation

Distribution of genotypes among both patients and controls was not deviated from Hardy Weinberg Equilibration as investigated by Arlequin 3.1.^19^ The Frequencies of each genotypes and alleles at 16C/A position of CCL22 gene is illustrated in table 2. CCL22 16 CC genotype was the wild type genotype and accounted for the most frequent genotype in both patients and control groups (112; 75.7% and 119; 80.4%, respectively). Mutant homozygous genotype (AA) was the least frequent with 3 (2.0%) in patients and 5 (3.4%) among controls (P>0.05). The frequency of CA genotype was 33 (22.3%) among patients and 24 (16.2%) among controls respectively. The minor allele frequency (MAF) was 0.12 in the whole population; with no significant different frequency when compared between patients and controls (39; 13.2% and 34; 11.5%, respectively, P=0.94). There were no significant differences in allele and genotype frequencies at 16C/A position of CCL22 between controls and lung cancer patients. No association was found between genotypes/alleles distribution and the type of lung cancer (table 2).

**Table 2:** Distribution of genotypes and alleles at the locus 16C/A in CCL22 gene and C1014T in CCR4 gene in patients with lung cancer and healthy controls

			**Controls ** **N=148, 2n=296**	**Patients N=148, 2n=296**	**P value⃰ **	**NSCLC ** **N=116, 2n= 232**	**P value□ **	**SCLC ** **N=29,** **2n=58**	**P value **
*CCL22* C16A (rs. 4359426)	Genotypes	CC	119 (84.4%)	112 (75.7%)	0.40	90 (77.6)	0.68	19 (65.5%)	0.13
		CA	24 (16.2%)	33 (22.3%)	0.24	24 (20.7)	0.44	9 (31%)	0.11
		AA	5 (3.4%)	3 (2.0%)	0.72	2 (1.7)	0.47	1(3.4%)	1.00
	Alleles	C	262 (88.5%)	257 (86.8%)	0.62	204 (87.9%)	0.94	47 (81.1%)	0.18
		A	34 (11.5%)	39 (13.2%)		28 (12.1%)		11 (18.9%)	
*CCR4* C1014T (rs. 2228428)	Genotypes	CC	80 (54.1%)	76 (51.4%)	0.73	61 (52.5%)	0.91	13 (44.8%)	0.48
		CT	49 (33.1%)	60 (40.5%)	0.23	48 (41.3%)	0.21	12 (41.3%)	0.52
		TT	19 (12.8%)	12 (8.1%)	0.25	7 (6.2%)	0.10	4 (13.9%)	0.1
	Alleles	C	209 (89.6%)	212 (71.6%)	0.85	170 (73.3%)	0.56	38 (65.5%)	0.54
		T	87 (29.4%)	84 (28.4%)		62 (26.7%)		20 (34.5%)	

□All in comparsion to control group. NSCLC: Non small cell lung cancer; SCLC: Small cell lung cancer

The distribution of CC, CT and TT genotypes at position C1014T of CCR4 gene was 76 (51.4%), 60 (40.5%) and 12 (8.1%) in patients and 80 (54.1%), 49 (33.1%) and 19 (12.8%) in the control group respectively. The minor allele frequency (MAF) was 0.29 in the whole population; with no significant different frequency when compared between patients and controls (84; 28.4% and 87; 29.4%, respectively, P=0.085). No differences were found in genotype/alleles frequencies between patients and controls. Statistical analysis revealed no considerable association between genotype/alleles frequencies of CCR4 C1014T SNP and the type of lung cancer (table 2).

## Discussion

Many studies have shown that genetic polymorphisms in genes coding chemokines and their receptors are associated with specific immunologic diseases and malignancies. In the current study we investigated the association between CCL22 gene polymorphism at position 16C/A, and CCR4 gene polymorphism at position C1014T and lung cancer. Results showed that alleles and genotypes of these two genetic polymorphisms are not differentially distributed between patients with lung cancer and the control group. Furthermore, no significant association was found between genotype frequencies of CCR4 and CCL22 in either NSCLC or SCLC types. The minor allele frequencies (MAFs) in the selected population were 0.12 and 0.29 respectively, at position 16 C/A in CCL22 gene and C1014T SNP in CCR4. 

The 16C/A SNP in CCL22 gene, causes an aspartate to alanine substitution in the CCL22 protein and likely the overexpression of CCL22.^16^ In a study by Wang et al. in Japan, an association between this polymorphism and susceptibility to gastric cancer was suggested both alone and in interaction with* H. pylori*.^16^ In a study conducted by Hirota et al. the relationship between CCL22 variants and atopic dermatitis was found in a group of Japanese patients.^20^ Despite the fact that the numbers of studies dealing with the CCL22 genetic variation in cancer is limited, some studies showed a statistically significant elevated level of CCL22 protein in malignancies such as Hodgkin lymphoma, colorectal adenocarcinoma and early stages of prostate cancer.^21^^-^^23^ Other studies revealed a link between higher CCL22 protein level in Sjogren’s syndrome and Hodgkin Lymphoma, a condition which is associated with poor prognosis of the diseases.^22^^,^^24^ In contrast, a research on the Japanese lung cancer patients revealed that the expression of CCL22 is associated with an increase in disease-free-survival and decrease in recurrence of cancer post-operation and better prognosis.^11^

In another study,^17^ on association was shown between CCL22 16C /A SNP and breast cancer nor with colorectal cancer (unpublished data) among the Iranian population. Disagreement between different studies regarding CCL22 genetic association with cancer might arises from mismatch in sample size and minor allele frequencies (MAFs) of the genetically different populations. Theses discrepancies however may come from the different in the etiopathology of cancers.

In the current study, the association between CCR4 single point mutation at locus C1014T with lung cancer was also not found. C1014T SNP causes a synonymous substitution in 338 Tyrosine of the CCR4 protein; therefore, while it does not affect the function of the protein but it has been suggested to change mRNA stability and further risk of malignancy.^18^ A study on the Japanese population showed lack of association of C1014T SNP with Atopic Dermatitis^18^ but in a study by Naeimi et al. this SNP was highly associated with gestational trophoblastic disease suggesting that this genotype can be used as a prognostic marker.^25^

Despite the number of studies dealing with CCR4 genetic variation and cancer is infrequent, huge array of studies linked CCR4 protein expression with cancer. The expression of CCR4 by tumor cells has been indicated to be related to the large-cell transformation of the common type of Cutaneous Non-Hodgkin T-cell lymphoma.^8^ In another study conducted by Lee et al. the overexpression of CCR4 was associated with tumor recurrence and a lower survival in patients with gastric cancer.^26^ In a study by Li et al. the correlation of CCR4 expression with HER2 expression, tumor recurrence and lymph node, lung and bone metastasis was revealed in breast cancer. This group indicated the strong association of CCR4 expression and lower overall survival.^27^ Another study by Nakamura et al. showed that differentiating osteoclasts which produce CCL22 can induce cell-migration of a human cancer cell-line SBC-5 that expresses CCR4 in mice model. This further suggests that overexpression of CCL22 in osteoclasts may promote bone metastasis in certain types of lung cancer.^28^

The association of CCR4 overexpression with diseases is not limited to cancer. Vestergaard et al. in Japan reported an increased CCR4/TARC expression in atopic dermatitis considered as a T-helper (Th) 2-dominated disease.^29^ Also, Rottman et al. postulated that increased CCR4 expression in psoriasis lesions may contribute to T lymphocyte trafficking to the dermis.^30^ Anti-CCR4 monoclonal antibodies (mAbs) have been recently recommended for the treatment of T-cell lymphomas, asthma and patients with CCR4-positive Hodgkin’s lymphoma.^9^^,^^31^^-^^34^

To the best of our knowledge, this is the first study that investigates the association between 16C/A SNP in CCL22 gene as well as C1014T SNP in CCR-4 gene and lung cancer. The data neither supports the association of the investigated polymorphisms with lung cancer nor with the type of tumor in the patients. While a limitation on the number of patients could be considered as a drawback of this study, its strength is within acceptable margins (64 for CCL22 and 70 for CCR4). 

## Conclusion

The results of this study demonstrates that CCL22 gene polymorphism at position 16C/A and CCR4 gene polymorphism at position C1014T, appear not to be associated with susceptibility to lung cancer. Multi-SNP analysis and haplotype deduction is required to completely eliminate the role of CCL22 and CCR4 genetic changes in susceptibility to lung cancer. 
